# Novel Simplified and Rapid Method for Screening and Isolation of Polyunsaturated Fatty Acids Producing Marine Bacteria

**DOI:** 10.1155/2012/542721

**Published:** 2012-08-15

**Authors:** Ashwini Tilay, Uday Annapure

**Affiliations:** Food Engineering and Technology Department, Institute of Chemical Technology, Matunga, Mumbai 400 019, India

## Abstract

Bacterial production of polyunsaturated fatty acids (PUFAs) is a potential biotechnological approach for production of valuable nutraceuticals. Reliable method for screening of number of strains within short period of time is great need. Here, we report a novel simplified method for screening and isolation of PUFA-producing bacteria by direct visualization using the H_2_O_2_-plate assay. The oxidative stability of PUFAs in growing bacteria towards added H_2_O_2_ is a distinguishing characteristic between the PUFAs producers (no zone of inhibition) and non-PUFAs producers (zone of inhibition) by direct visualization. The confirmation of assay results was performed by injecting fatty acid methyl esters (FAMEs) produced by selected marine bacteria to Gas Chromatography-Mass Spectrometry (GCMS). To date, this assay is the most effective, inexpensive, and specific method for bacteria producing PUFAs and shows drastically reduction in the number of samples thus saves the time, effort, and cost of screening and isolating strains of bacterial PUFAs producers.

## 1. Introduction

Microbial lipids are a diverse group of compounds with a number of vital nutraceutical and pharmaceutical applications and utilized commercially since the 1980s. These microbial lipids or polyunsaturated fatty acids (PUFAs) are obtained from various sources. Now a day, microorganism-produced (algae/fungi/bacteria) PUFAs are commercially competitive with plant and fish oils.

PUFAs are the fatty acids having more than one double bond. Eicosapentaenoic acid (EPA, 20 : 5, *n*-3) and docosahexaenoic acid (DHA, 22 : 6, *n*-3) are the important *n*-3 fatty acids, while arachidonic acid (AA, 20 : 4, *n*-6) is a vital *n*-6 fatty acid. EPA and DHA are important for prevention of arthrosclerosis, cancer, rheumatoid arthritis, psoriasis, and diseases of old age such as Alzheimer's and age-related macular degeneration [1, 2]. AA and DHA are of special importance in the brain and blood vessels and are considered essential for pre- and postnatal brain and retinal development [3]. Eicosanoids such as prostaglandins, prostacyclins, and leukotrienes derived from *n*-3 PUFA are also important in new-born and infant development, modulatory vascular resistance, and wound healing [4–6]. PUFAs are either directly available as components of the diet or produced from precursors like linoleic acid (LA, C18 : 2 *n*-6) and a-linolenic acid (ALA, C18 : 3 *n*-3) [7].

Accordingly, PUFAs are highly important substances in the pharmaceutical, medical, and nutritional fields. Recent investigations have focused on microorganisms as alternative natural source for production of oil containing PUFAs. These are potentially promising lipid source because of their high growth rates in simple media and simplicity of their manipulation. As traditional sources of *n*-3 fatty acids such as fish oil continue to diminish, identification of alternate sources will become crucial. Marine microorganisms represent one of the less explored sources of biologically active natural products. Novel compounds with various bioactivities such as antibiotic, antitumor, cytotoxic, and anti-inflammatory, have been isolated and elucidated from this source [8].

Considering importance of PUFAs, many researchers have tried to isolate and screen marine organisms for these bioactive compounds. The next step after isolation of microorganisms is screening. Ideally, selective procedure would allow the detection and isolation of microorganisms producing the desired metabolite. This primary screening should be rapid, inexpensive, predictive, specific, but effective over a broad range and should be applicable to large scale. Sometimes primary screening is time consuming and labour intensive when a large number of isolates have to be screened to identify a few potential ones. However, this is possibly the most critical step since it eliminates the large bulk of unwanted isolates, which are either nonproducers or producers of known compounds.

Screening and isolation method for long chain PUFA by marine protistan was reported but judged unsuitable by Bowles et al. for high throughput screening. The H_2_O_2_ plate assay method can be applied to a wide range of bacterial samples collected from different regions or from different animal sources. During screening of random hundreds of different marine bacterial samples, we have successfully discovered new strains of marine microorganisms with the special characteristic to produce PUFAs.

**Figure 1:**
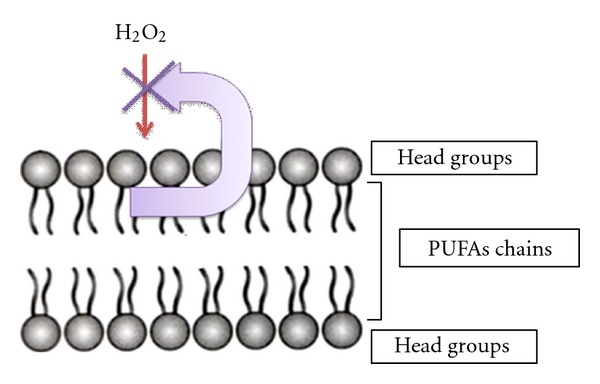
Proposed mode of action for the H_2_O_2_-plate assay method.

Generally, PUFAs are the molecules which are most susceptible to oxygen and reactive oxygen species (ROS) [10]. There are facts that PUFAs are stable when they are in vivo against oxidative stresses caused by ROS. This study was based on application of antioxidative effect of PUFA against ROS (Figure 1), for rapid screening of large number of marine isolates. However, no information regarding the screening of PUFAs producing marine bacteria has been reported. In the following strategy, we have presented qualitative method for rapid screening of PUFA producers.

## 2. Experimental Procedures

### 2.1. Materials

All the chemicals and media components used in the present study were AR grade and purchased from Hi Media Ltd, Mumbai, India. Sodium azide was purchased from S.D. Fine Chemicals Limited, Mumbai, India.

### 2.2. Media and Culture Conditions for Marine Microorganisms

#### 2.2.1. Sample Collection

**Figure 2:**
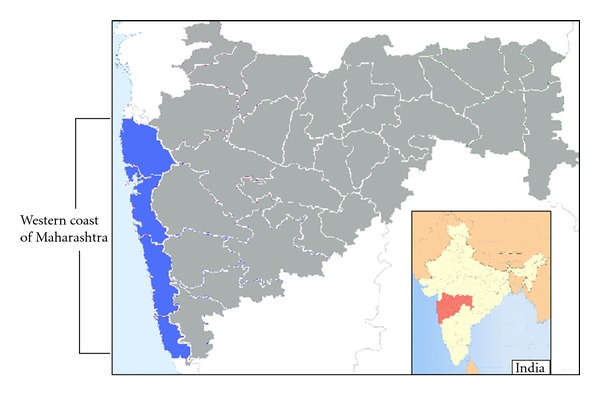
Map of western coast region of Maharashtra (INDIA).

Various samples were collected from different regions of western coast of Maharashtra, India (Figure 2) and were brought to our laboratory for further investigation.

#### 2.2.2. Isolation of Marine Bacteria

The collected samples were serially diluted and plated over nutrient agar plates. The inoculated plates were incubated at 28 ± 2°C for 24–48 h. After incubation, the selected bacterial colonies were purified and subcultured on nutrient agar medium for further investigation. All the marine isolates were preserved in glycerol stocks under (−) 20°C.

### 2.3. Primary Screening of Marine Bacterial Isolates by H_2_O_2_-Plate Assay

**Figure 3:**
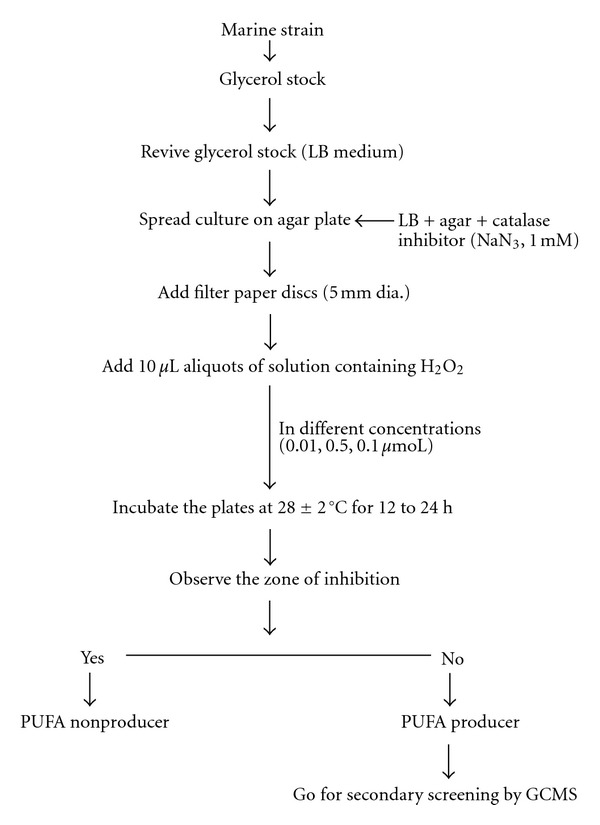
Protocol for primary screening of marine isolates.

About 100 isolated marine strains were randomly selected and screened for PUFA production (Figure 3). All the selected marine cultures were cultivated in Luria-Bertani (LB) medium (1% Tryptone, 0.5% Yeast Extract, 1% NaCl) at 28 ± 2°C for 24 h, 180 rpm. To this medium, 0.5% NaCl was added for proper growth of marine isolates rather than using artificial seawater [11]. To find out response of bacteria to H_2_O_2_, bacterial culture reaching an optical density at 660 nm of 1.0 was used and spreaded over plate containing LB medium and sodium azide (NaN_3_, 1 mM). On a surface filter paper discs of diameter 5 mm were placed and ten microliter aliquots of solution containing different concentration of H_2_O_2_ (0.01, 0.5, 1.0% prepared from 30% stock solution) were added on filter disc. Plates were then incubated at 28 ± 2°C for 24 h. Zone of inhibition was observed and further confirmation of PUFA producers was done by GCMS.

### 2.4. Secondary Screening and Confirmation

#### 2.4.1. Media and Cultivation Conditions

Amongst all screened marine bacterial cultures, the positive strains were selected on the basis of no zone of inhibition (PUFA producers) and used further for secondary screening and confirmation for the production of PUFA. A loopful of the bacterial culture grown on previously preserved LB medium slants was transferred into 20 mL of LB broth in a 100 mL Erlenmyer flask and left on a shaker at 28 ± 2°C for 24 h at 180 rpm. One mL of culture was transferred into 50 mL fresh sterilized LB broth and left on shaker at 28 ± 2°C for 24 h at 180 rpm. Cell biomass was harvested from fermented media by centrifugation at 8000 rpm at 25 ± 2°C for 10 min. The biomass was washed thoroughly with distilled water and was finally dried at 50°C overnight.

#### 2.4.2. Direct Lipid Extraction and Esterification of Fatty Acids

A rapid direct extraction and esterification method was developed and applied in this study. Extraction of lipids from biomass was performed according to the modified procedure of Hoshi et al. [12]. Lipids were extracted for 3-4 h with a 3 times volume of chloroform/methanol (2 : 1, v/v) containing 15 mg BHT for prevention of oxidation of PUFAs [13]. Esterification was done by adding 0.2 mL of 20 mM cupric acetate monohydrate in methanol and 1 mL of 0.5 N HC1 in methanol was added, and the mixture was left for the specified time (2-3 h) at room temperature or at 28 ± 2°C. The reaction was stopped with the addition of 0.4 mL of water. The lower chloroform layer was pooled and then evaporated and was concentrated by rotary evaporator (Buchi Rotavapor) at 35°C. Finally the fatty acid methyl esters (FAME) were dissolved in hexane (1 mL), filtered through Whatman No. 1 filter paper, and analysed by GCMS.

#### 2.4.3. Analysis PUFA by GCMS

Analysis of the FAME was performed by GCMS using slightly modified procedure [14]. All compounds were identified by comparison of their retention times with those of known standards and confirmed by GCMS using Varian 220-MS ion trap mass spectrometer (Varian, Inc. Walnut Creek, CA) connected to Varian 450-GC equipped with CP-SIL 88 capillary column (25 m × 0.25 mm i.d. × 0.39 mm OD, Varian). The injector was maintained at 250°C, and the column oven was programmed to increase from 160 to 220°C at 7°C min and then maintained at 220°C for 10 min. Split ratio was adjusted to 1 : 20. Helium was used as the carrier gas and flow rate was maintained at 1 mL/min. GCMS was operated at an ionization voltage of 70 eV and trap temperature at 220°C with mass range of 40–350 atomic mass units.

## 3. Results and Discussion

### 3.1. Primary Screening of Marine Bacterial Isolates by H_2_O_2_-Plate Assay

**Figure 4:**
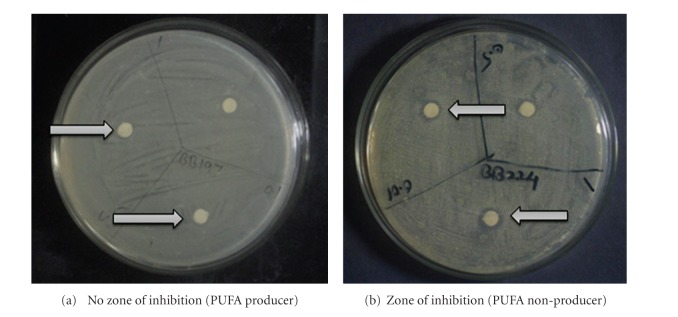
H_2_O_2_-plate assay.

In H_2_O_2_-plate assay method, the cells which are susceptible to externally-added H_2_O_2_ cannot able to grow suitably and thus shows a zone of inhibition which is dependant on added concentration of H_2_O_2_ on filter paper disc. The diameter of zone of inhibition is directly proportional to the concentration of added H_2_O_2_. The contradictory situation was observed for bacterial cells which produce PUFA. These cells able to grow in presence of added H_2_O_2_ on filter paper disc. As shown in Figure 4(a) the bacteria were grown even in presence of H_2_O_2_, due to the membrane-shielding effects of PUFAs. In most cases, PUFAs are among the molecules most vulnerable to oxygen and ROS, Okuyama et al. [10]. In Figure 4(b) the bacterial cells which were PUFA deficient or nonproducers were hampered by H_2_O_2_ and hence could not able to grow where zone of inhibition was directly proportional to the concentration of H_2_O_2_ added. Higher the concentration of H_2_O_2_ more will be the zone of inhibition.

To confirm that, growth of bacteria in presence of H_2_O_2_ is in reality mainly due to presence of PUFA; NaN_3_ was added in to the media which is a very powerful inhibitor of catalase. If microorganism is producing catalase enzyme, NaN_3_ inhibits catalase enzyme [15] which helps in interfering and promotes the actual interpretation of plate assay. About 1 mM concentration of NaN_3_ was used during experimental study. The concentration should be an adequate amount to act as catalase inhibitor at the same time should not be antimicrobial in nature. The concentration of NaN_3_ was decided from reported studied by Teixeira and Mota [16]. Out of selected 100 strains, 26 strains were found to give false positive results (Tables 1a and 1b). Out of 26, 10 strains were selected which gave false positive results at all H_2_O_2_ concentrations used during plate assay method. They were screened for further secondary analysis and confirmation.

EPA- and DHA-expressing bacteria were reported to be more resistant to exogenous H_2_O_2_ [10]. But there were no investigations whether other long chain (LC) PUFAs than EPA and DHA have similar effects. The bacterial cell gets protected by the effect of EPA that has reported earlier [10]. The membrane-shielding effects of *n*-3 LC-PUFAs have been shown only for bacterial cells producing EPA [17, 18]. From the study we have observed that other than EPA and DHA other PUFA might be responsible for the same protecting effect of exogenous H_2_O_2_ and our hypothesis was well supported with studied carried out by Okuyama et al. [10].

**Figure 5:**
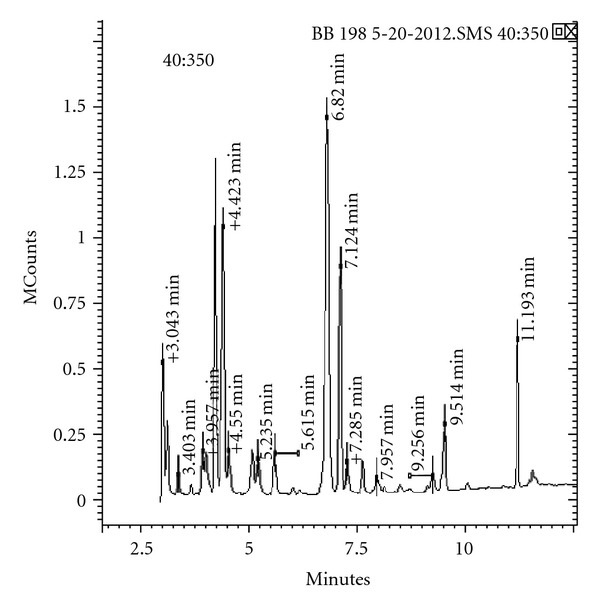
Gas chromatogram showing fatty acid profile of a selected marine isolate after primary screening.

As shown in Figure 5, the fatty acid profile obtained from the bacterial culture which was able to grow in presence of H_2_O_2_ shows mainly AA production along with EPA. Hence, from this study not only EPA and DHA but also other PUFAs like AA act as the shield molecules against such oxidative challenges exogenously and endogenously raised in marine environments. There are some reports on protective effect of DHA against the external hydrogen peroxide [19]. It was reported that PUFAs including *n*-3 PUFAs are the molecules which are most susceptible to oxygen and reactive oxygen species (ROS). But from our study, it was observed that not only *n*-3 PUFAs are susceptible to oxygen and reactive oxygen species but also *n*-6 PUFAs like AA may behave in same way [20].

### 3.2. Secondary Screening and Confirmation

From few selected false positive strains all strains were found to give remarkable response when their lipid extract was injected into GCMS. The mass spectra of all selected strains were found to produce different kind of fatty acids. Some of them were found to produce AA and EPA which are very essential PUFAs from nutritional point of view. The fatty acid profile of 10 selected strains was shown in Table 2 that provides the information about different fatty acids produced by selected marine bacteria after primary screening using H_2_O_2_-plate assay. Hence this method is a qualitative estimation of PUFAs produced from microorganisms.

## 4. Conclusion

Thus, the present investigation has clearly revealed the presence of PUFAs in marine bacteria in the marine samples by newly developed novel simplified and rapid plate culture method for screening of PUFAs-producing marine bacteria; collected from different regions of western coast of Maharashtra, India. GCMS analysis studies confirmed the actual production of PUFA in various selected marine bacteria after primary screening. In order to minimise the time required for analysis as well as economic loss, this method gives the suitable solution for large number of samples screening which are abundant in the marine environment.

## Figures and Tables

**Table:** (a)

No.	Strain	H_2_O_2_^@^ (%)			PUFA*	No.	Strain	H_2_O_2_^@^ (%)			PUFA*
		0.1	0.5	1.0	+ve/−ve			0.1	0.5	1.0	+ve/−ve
1	JAY B 8	−	−	−	−ve	26	TH B 9	−	−	−	−ve
2	TAV B 31	−	−	−	−ve	27	DI BB 152	−	−	+	+ve
3	PA BB 8	−	−	−	−ve	28	**TH BB 21**	**+**	**++**	**++**	**+ve**
4	TH BB 31	−	−	−	−ve	29	ALI B 71	−	−	−	−ve
5	DIG BB 113	−	−	−	−ve	30	R BB 11	+	+	−	+ve
6	TH BB 22	−	−	−	−ve	31	BB 82	−	−	−	−ve
7	PA BB 3	−	−	−	−ve	32	JAY B 12	−	−	−	−ve
8	TH BB 15	−	−	−	−ve	33	TH BB 22	−	−	−	−ve
9	P BB 13	−	−	−	−ve	34	MAN BB 358	−	−	−	−ve
10	**DIG BB 4**	**+**	**+**	**+**	**+ve**	35	P BB 9	−	−	−	−ve
11	**M BB 318**	**−**	**+**	**++**	**+ve**	36	TH 33	−	−	−	−ve
12	DI BB 146	−	−	+	+ve	37	R 23	−	−	−	−ve
13	GP B 2	−	+	++	+ve	38	RAT B 18	−	−	−	−ve
14	AH B 53	−	+	++	+ve	39	RA 21	−	−	−	−ve
15	**MD BB 345**	**+**	**+**	**++**	**+ve**	40	MD BB 325	−	−	−	−ve
16	PA BB 8	−	+	++	+ve	41	SHI BB 2	−	−	−	−ve
17	TH BB 5	−	+	+	+ve	42	PA B 12	−	−	−	−ve
18	MAN BB 200	−	−	−	−ve	43	PA BB 4	−	−	−	−ve
19	MAN BB 361	−	−	−	−ve	44	TH BB 27	−	−	−	−ve
20	AL BB 156	−	+	++	+ve	45	TH BB 25	−	−	−	−ve
21	TH BB 20	−	+	++	+ve	46	**BB 227**	**+**	**+**	**++**	**+ve**
22	**TAV B 27**	**+**	**+**	**++**	**+ve**	47	TH BB 30	−	−	−	−ve
23	**ALI B 51**	**+**	**+**	**++**	**+ve**	48	MAN 10	−	−	−	−ve
24	BB 232	−	+	+	+ve	49	BB 72	−	−	−	−ve
25	MAN 353	−	−	+	+ve	50	R 37	−	−	−	−ve

^@^describes zone of inhibition due to presence H_2_O_2_; +/++  describes growth of microorganisms or no zone of inhibition due to presence of PUFA.

*PUFA +ve denotes PUFA producer and −ve denotes PUFA nonproducer.

Note: highlighted strains were further screened by secondary analysis.

**Table:** (b)

No.	Strain	H_2_O_2_^@^ (%)			PUFA*	No.	Strain	H_2_O_2_^@^ (%)			PUFA*
		0.1	0.5	1.0	+ve/−ve			0.1	0.5	1.0	+ve/−ve
51	**TH BB 19**	**++**	**++**	**+**	**+ve**	76	MD BB 312	−	−	−	−ve
52	**BB 83**	**+**	**+**	**+**	**+ve**	77	MAN BB 63	−	−	−	−ve
53	RAT B 3	−	−	−	−ve	78	TH BB 28	−	−	−	−ve
54	AK BB 189	−	−	−	−ve	79	JAY B 38	−	−	−	−ve
55	BB 201	−	−	−	−ve	80	BB 20	+	−	−	+ve
56	MAN 352	−	−	−	−ve	81	P BB 22	−	−	−	−ve
57	TH 33	+	−	−	+ve	82	RAT B 85	−	−	−	−ve
58	**BB 198**	**++**	**+**	**+**	**+ve**	83	SHI BB 2	−	−	−	−ve
59	R 13	−	−	−	−ve	84	PA BB 33	−	−	−	−ve
60	HAR BB 118	−	−	−	−ve	85	MD BB 122	−	−	−	−ve
61	RO BB 24	+	−	−	+ve	86	MAN BB 229	+	−	−	+ve
62	TH BB 24	−	−	−	−ve	87	TH BB 82	−	−	−	−ve
63	SRK 6	−	−	−	−ve	88	DIG BB 122	−	−	−	−ve
64	BB 245	−	−	−	−ve	89	G BB 89	−	−	−	−ve
65	BB 86	−	−	−	−ve	90	MAN 63	−	−	−	−ve
66	G BB 249	−	−	−	−ve	91	AL BB 148	−	−	−	−ve
67	DIG BB 108	−	−	−	−ve	92	TAV B 47	−	−	−	−ve
68	MD BB 338	−	−	−	−ve	93	R 39	−	−	−	−ve
69	TH BB 166	−	−	−	−ve	94	BB 289	+	−	−	+ve
70	MAN BB 364	−	−	−	−ve	95	JAY B 23	−	−	−	−ve
71	SRK 23	−	−	−	−ve	96	DI BB 278	−	−	−	−ve
72	TH BB 83	−	−	−	−ve	97	TH B 34	−	−	−	−ve
73	BB 39	−	−	−	−ve	98	AK BB 148	−	−	−	−ve
74	TAV B 29	−	−	−	−ve	99	HAR BB 72	−	−	−	−ve
75	SHE BB 29	−	−	−	−ve	100	RO BB 41	−	−	−	−ve

^@^describes zone of inhibition due to presence H_2_O_2_; +/++ describes growth of microorganisms or no zone of inhibition due to presence of PUFA.

*PUFA +ve denotes PUFA producer and −ve denotes PUFA nonproducer.

Note:  highlighted strains were further screened by secondary analysis.

**Table 2:** Fatty acid profile of marine isolate produced after fermentation in secondary screening.

Fatty acid methyl esters (MUFA + PUFA)	Selected strains for secondary screening and their fatty acid profile									
	M BB 318	BB 83	ALI B 51	BB 227	MD BB 345	BB 198	DIG BB 4	TAV B 27	TH BB 19	TH BB 21
Pentadecanoic acid	**+**	**+**	**+**	**+**	**+**	**+**	**−**	**−**	**−**	**−**
Hexadecanoic acid	**+**	**+**	**+**	**+**	**+**	**+**	**+**	**+**	**+**	**+**
14-methylhexadecanoate	**+**	**−**	**+**	**+**	**+**	**+**	**−**	**−**	**−**	**−**
Margaric acid	**+**	**+**	**+**	**+**	**+**	**+**	**−**	**−**	**−**	**−**
Oleic acid	**+**	**+**	**+**	**+**	**+**	**+**	**−**	**+**	**+**	**+**
16-Octadecenoic acid	**−**	**−**	**−**	**−**	**−**	**−**	**+**	**−**	**−**	**−**
7,10-Octadecadienoic acid	**−**	**−**	**−**	**−**	**−**	**−**	**+**	**−**	**−**	**−**
Linoleic acid	**+**	**+**	**+**	**+**	**+**	**+**	**−**	**+**	**+**	**+**
7,10,13-Hexadecatrienoic acid	**−**	**−**	**−**	**−**	**−**	**−**	**+**	**−**	**−**	**−**
*γ*-Linolenic acid	**−**	**−**	**−**	**−**	**−**	**+**	**−**	**−**	**−**	**−**
Linolenic acid	**+**	**+**	**+**	**+**	**+**	**+**	**−**	**+**	**+**	**+**
Eicosanoic acid	**+**	**−**	**−**	**−**	**−**	**−**	**−**	**−**	**−**	**−**
Arachidonic acid	**−**	**−**	**−**	**−**	**−**	**+**	**−**	**−**	**−**	**−**
Eicosapentaenoic acid	**−**	**−**	**−**	**−**	**−**	**+**	**−**	**−**	**−**	**−**

Note: MUFA- monounsaturated fatty acid.
